# Pan-Cancer Analysis of the COVID-19 Causal Gene *SLC6A20*

**DOI:** 10.1021/acsomega.3c00407

**Published:** 2023-03-31

**Authors:** Ahmet Acar

**Affiliations:** Department of Biological Sciences, Middle East Technical University, Universiteler Mah. Dumlupınar Bulvarı 1, Çankaya, Ankara 06800, Turkey

## Abstract

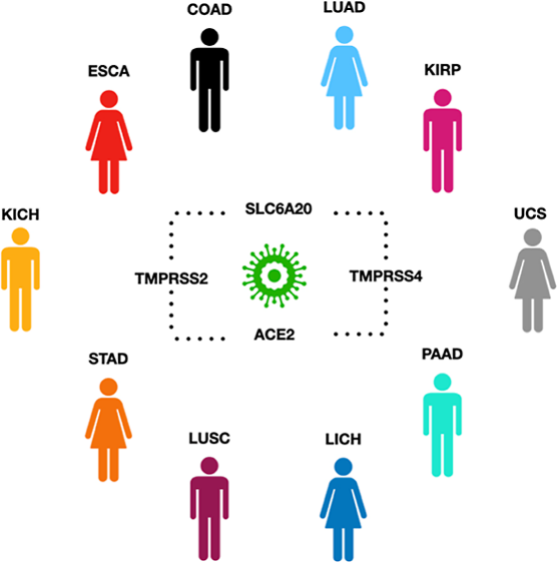

Genome-wide association studies demonstrated that the chromosome 3p31.21 locus was linked to the severity of COVID-19 disease. The *SLC6A20* gene was reported to be one of the critical causal genes regulated by this locus. Various studies focused on demonstrating the severity of COVID-19 in cancer patients and reported that elevated SARS-CoV-2-associated gene expression might contribute to increased susceptibility for COVID-19 in cancer patients. Given that pan-cancer association for the COVID-19 causal gene *SLC6A20* is lacking, we aimed to perform systematic profiling of *SLC6A20* in different malignancies. Human Protein Atlas, UALCAN, and Hepatocellular Carcinoma (HCCDB) databases were used to assess *SLC6A20* gene expression changes in The Cancer Genome Atlas samples with respect to their normal counterparts. GEPIA and TIMER2.0 databases were used to determine the correlation between *SLC6A20* and COVID-19-associated genes. Different databases were used for identification of the correlation of *SCL6A20* with infiltrating immune cells. The canSAR database was utilized to determine the association of *SCL6A20* with immune profiling in different malignancies. The STRING database was utilized to determine the protein network interacting with *SLC6A20*. Here, we showed *SLC6A20* mRNA expression in pan-cancer samples and their normal counterparts. Increased *SCL6A20* expression was associated with tumor grade, and there was a positive correlation with SARS-CoV-2-associated genes. Furthermore, *SLC6A20* expression was positively correlated with infiltrating neutrophils and immune-related signatures. Lastly, *SLC6A20* expression was found to be associated with the angiotensin converting enzyme 2 homologue, TMEM27, suggesting a potential link between *SLC6A20* and COVID-19. Taken together, these results suggest that elevated *SLC6A20* levels might be partly responsible for increased susceptibility of cancer patients to COVID-19 disease. Therapeutic intervention strategies against *SLC6A20* in cancer patients, alongside other treatment modalities, might offer a benefit in delaying COVID-19 disease.

## Introduction

SARS-CoV-2 outbreak started in 2019, and the World Health Organization (WHO) declared a pandemic in 2020. As of 30 December 2022, there have been approximately 663,380,366 reported cases with a total of 6,691,567 deaths (https://www.worldometers.info/coronavirus/).

Viral envelope-mediated or direct fusion of the virus with cell membrane is facilitated by certain proteases whereby the viral spike protein (S1) is activated.^1^ The interaction between the SARS-CoV-2 spike protein and the angiotensin converting enzyme 2 (ACE2) receptor mediates the entry of the virus into a cell.^2^ Upon this interaction, protease cleavage within the spike protein is initiated. Within this process, various proteases including cathepsin L, cathepsin B, furin, and transmembrane protease serine 2 (TMPRSS2) are involved.^3^ TMPRSS2 is one of the critical proteases in this process, activating the S protein for the entry of the virus into a cell.^4^ Following the release of viral genome into the host cell, viral replicase proteins are translated, and subsequently these replicase proteins initiate the viral replication of genomic RNA.^5^ Finally, the genomic RNA is translated into structural protein subunits of virus particles.^6^

Cancer is a complex inflammatory disease and linked to an impaired immune system.^7−10^ Several studies have shown that cancer patients can be more susceptible to SARS-CoV-2 infection than the general population.^11,12^ Although in-depth understanding of the susceptibility of cancer patients to COVID-19 is still unclear, COVID-19-associated genes, ACE2, TMPRSS2, and TMPRSS4 were upregulated and co-expressed in different cancer types.^13−15^ In addition to pan-cancer studies, COVID-19 susceptibility in cancer patients was explained with elevated levels of endosomal entry and recycling proteins for SARS-CoV-2 infection.^16^ Given the accumulating evidence documenting COVID-19 susceptibility in cancer patients, characterizing additional genes in these patients could contribute to identifying new targets.

Genome-wide association studies (GWASs) identified host-specific genetic factors contributing to COVID-19.^17,18^ Among these, it was reported that chromosome 3p21.31 is associated with COVID-19^18^ Therefore, the chromosome 3p21.31 locus was investigated to delineate the causal gene for COVID-19 in this locus using both genome and epigenome editing.^19^ The *SLC6A20* gene was identified as one of the causal genes in the chromosome 3p21.31 locus for the severity of COVID-19.^19^ In addition, it was demonstrated that *SLC6A20* gene participates as a putative causal gene for the severity of COVID-19, using a genome-wide clustered regularly interspaced short palindromic repeats (CRISPR) loss-of-function study.^20^ Given the contribution of *SLC6A20* gene in COVID-19 severity and increased COVID-19 susceptibility observed in cancer patients, it was aimed to study the *SLC6A20* expression profile and association with SARS-CoV-2 infection genes and immune modulation in pan-cancer data sets.

Here, we show systematic profiling of *SLC6A20* expression in pan-cancer tumor and healthy samples together with correlation with SARS-CoV-2 infection genes ACE2, TMPRSS2, and TMPRSS4 and immune filtration in tumor samples. The *SLC6A20* interacting protein TMEM27, an ACE2 homologue, and its pan-cancer expression profiling analysis suggests the involvement of *SLC6A20* in providing increased susceptibility of COVID-19 disease in cancer patients. These findings suggest a potential therapeutic intervention strategy for *SLC6A20* in cancer patients with the COVID-19 disease.

## Results

### *SLC6A20* Expression in Healthy Samples

**Figure 1:**
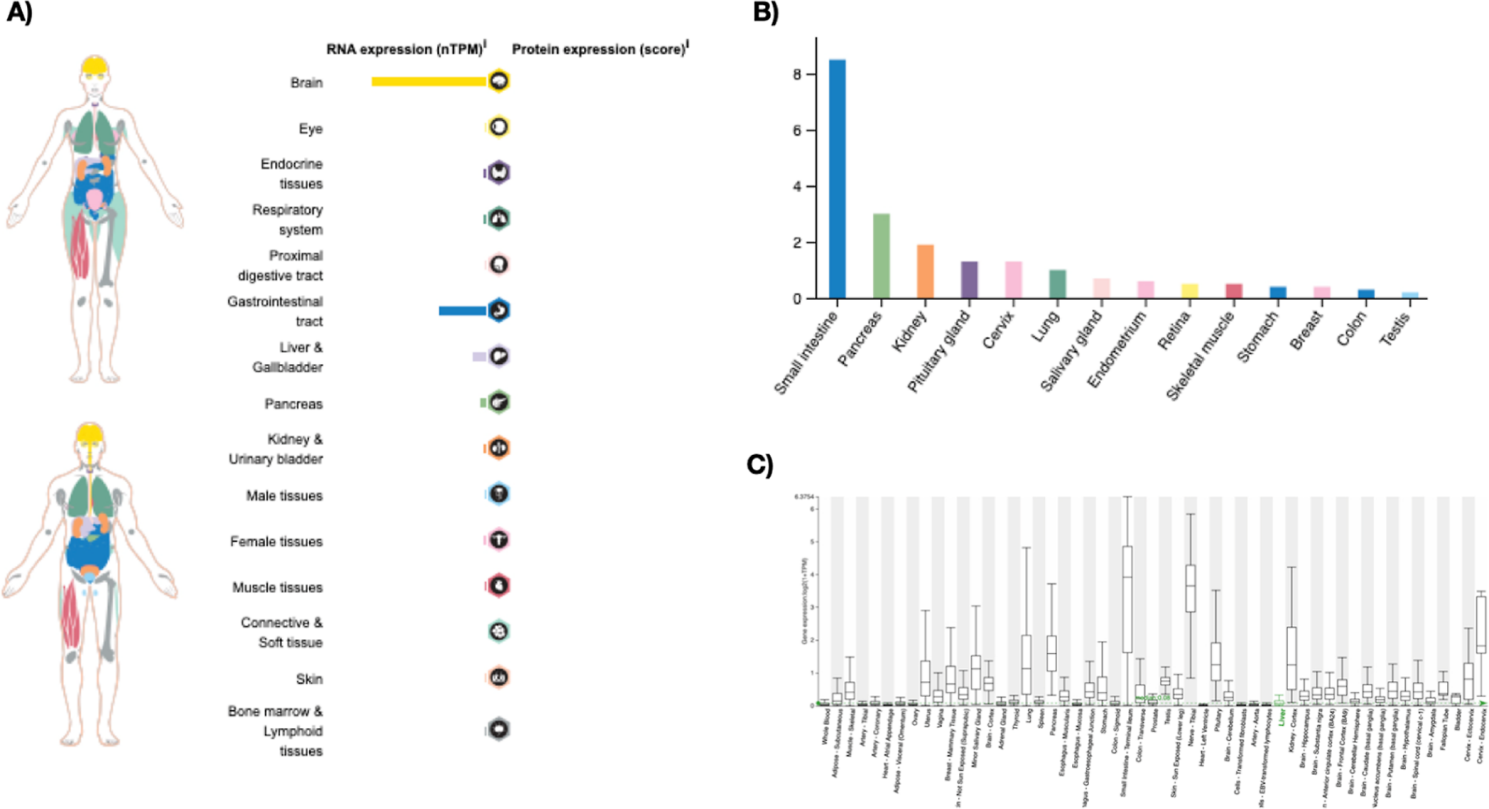
(A) Normal mRNA expression of *SLC6A20* was analyzed using the HPA database. (B) *SLC6A20* mRNA expression profile in normal tissues and organs from the GTEx database is presented. (C) *SLC6A20* gene expression in various normal tissues was analyzed using the HCCDB database.

To investigate the tissue-specific *SLC6A20* gene expression, a Human Protein Atlas (HPA) data set was explored. *SLC6A20* gene was predominantly expressed in brain, gastrointestinal track, liver and gallbladder, and pancreas more than other tissues (Figure 1A). Next, a GTEx data set from HPA was assessed, and *SLC6A20* expression was detected at high levels at small intestine, pancreas, and kidney (Figure 1B). Finally, using the Hepatocellular Carcinoma (HCCDB) database,^21^*SLC6A20* expression was confirmed in a number of healthy tissues. The HCCDB database demonstrated that *SLC6A20* was abundant in small intestine and nerve tissue, followed by kidney tissue (Figure 1C). Collectively, these data show that *SLC6A20* is expressed in a variety of normal tissues.

### Expression Profile of *SLC6A20* in Pan-Cancers

**Figure 2:**
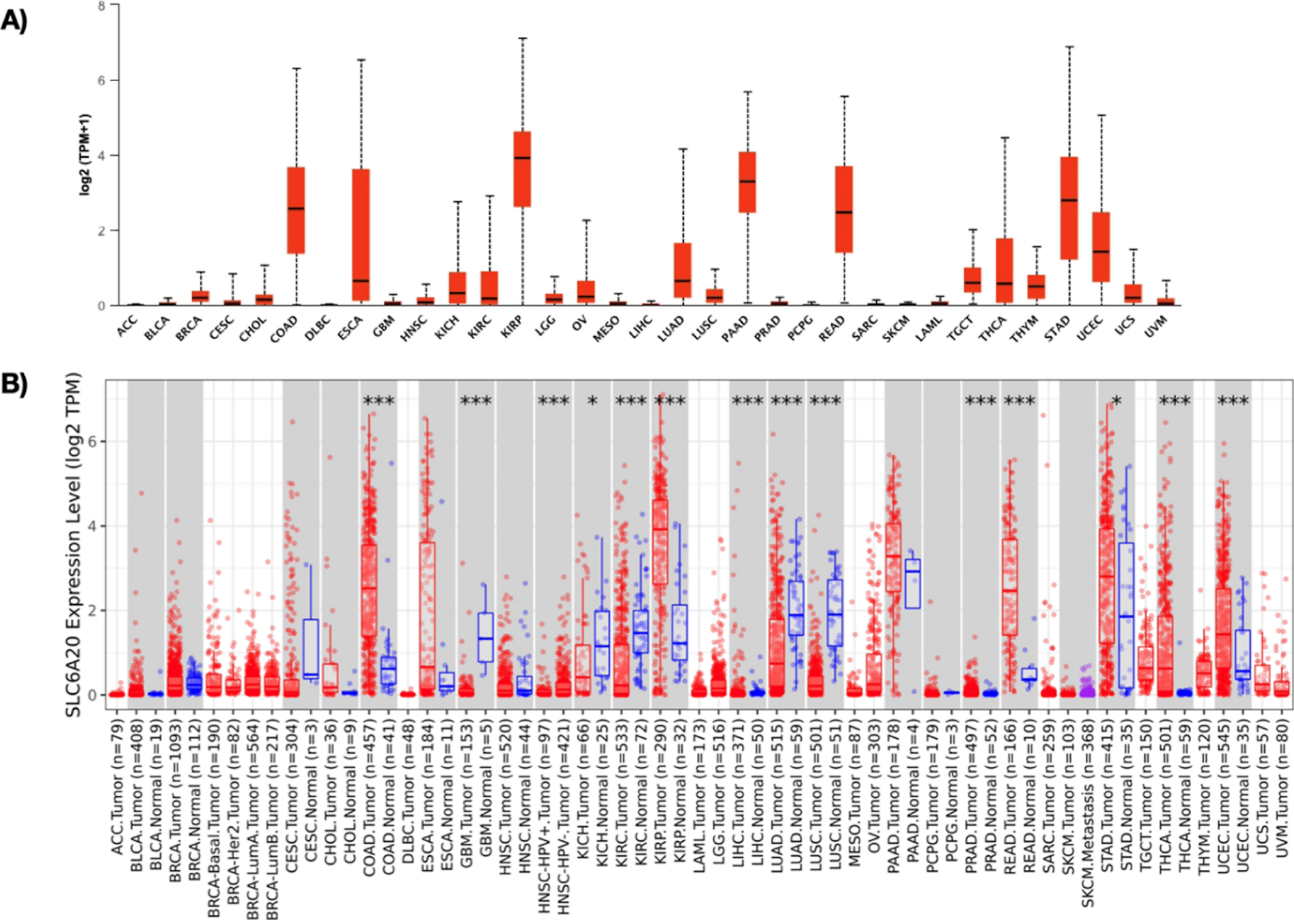
(A) *SLC6A20* mRNA expression profile in different malignancies without normal pairs was analyzed. (B) *SLC6A20* mRNA expression pan-cancer tumor samples with normal pairs was analyzed using UALCAN database. *P < 0.5, **P < 0.01, ***P < 0.001.

Using the pan-cancer The Cancer Genome Atlas (TCGA) data set, the *SCL6A20* gene expression profile was investigated. Between the TCGA tumor samples, *SCL6A20* was highly expressed in pancreatic adenocarcinoma (PAAD), kidney renal papillary cell carcinoma (KIRP), rectal adenocarcinoma (READ), stomach adenocarcinoma (STAD), and colon adenocarcinoma (COAD) (Figure 2A). Next, *SCL6A20* expression in different malignancies compared with normal samples was examined using UALCAN database^22^ (Figure 2B). *SCL6A20* was found to be elevated in COAD (*p* < 0.001), KIRP (*p* < 0.001), liver hepatocellular carcinoma (LIHC) (*p* < 0.001), prostate adenocarcinoma (PRAD) (*p* < 0.001), READ (*p* < 0.001), STAD (*p* < 0.05), thyroid carcinoma (THCA) (*p* < 0.001), and uterine corpus endometrial carcinoma (UCEC) (*p* < 0.001) compared with the expression in healthy samples (Figure 2B). On the other hand, *SLC6A20* expression was downregulated in glioblastoma multiform (GBM), kidney chromophobe (KICH), kidney renal clear cell carcinoma (KIRC), lung adenocarcinoma (LUAD), and lung squamous carcinoma (LUSC) samples when compared to normal (Figure 2B). Taken together, these findings indicated that *SLC6A20* was predominantly expressed in gastrointestinal tumors, which is one of the most frequently seen sites for SARS-CoV-2 infection in cancer patients.^14,23,24^

### *SLC6A20* Expression Might Predict High Tumor Grade

**Figure 3:**
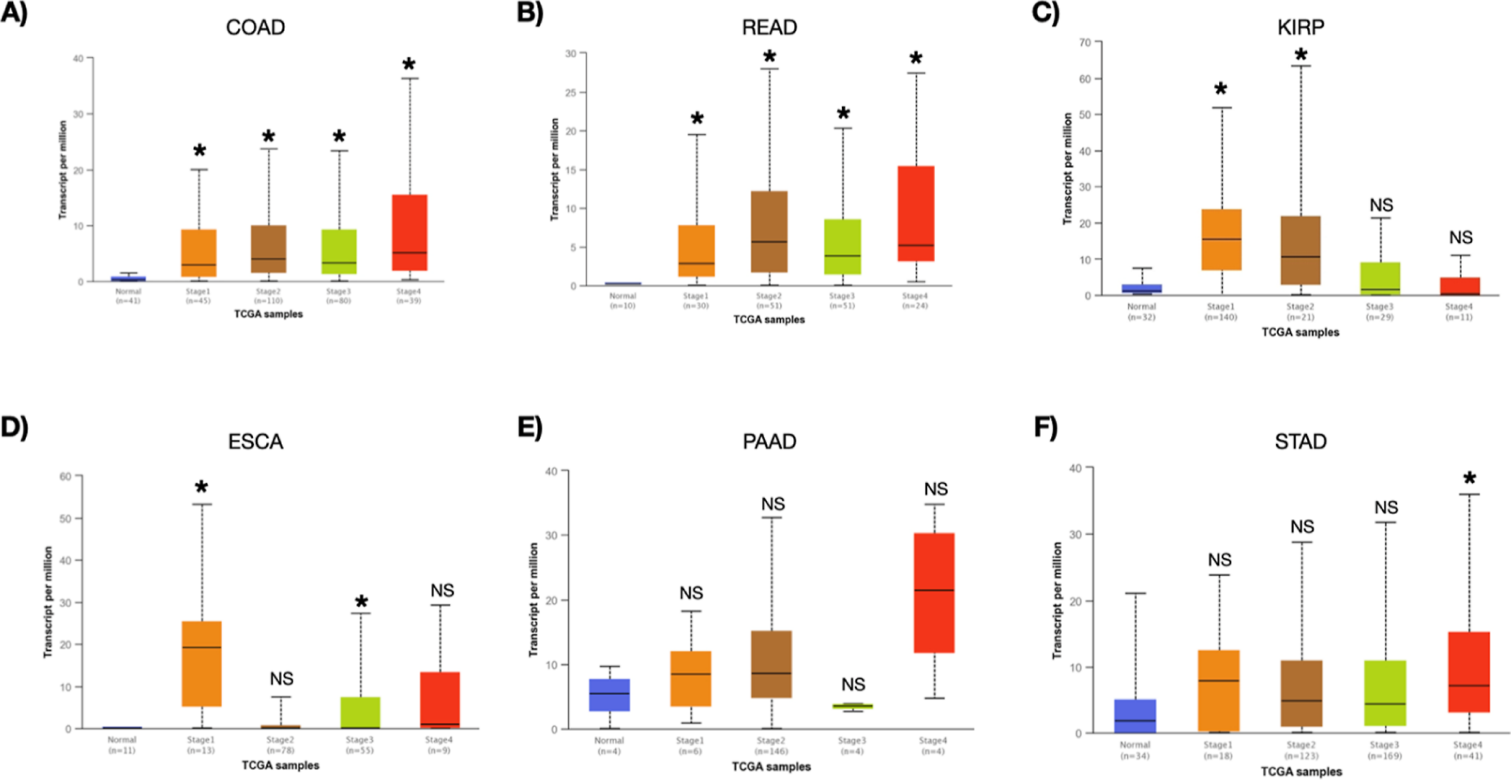
*SLC6A20* gene expression in TNM stages (stage 1, 2, 3, and 4) in (A) COAD, (B) READ, (C) KIRP, (D) ESCA, (E) PAAD, and (F) STAD samples from UALCAN database (* denotes that *p*-value is less than 0.05, and NS means no significance when compared to their respective control samples).

Given the high expression of *SLC6A20* gene in a variety of human cancers, the UALCAN database was utilized. Expression of *SCL6A20* was found to be statistically higher in stage 1-to-4 in COAD and READ samples compared to normal samples (Figure 3A,B). In KIRP, *SCL6A20* expression was found to be higher only in stage 1 and stage 4 samples in comparison to normal samples, while the mRNA expression of *SCL6A20* in stage 2 and stage 3 samples was not significantly upregulated (Figure 3C). Furthermore, elevated *SCL6A20* expression in ESCA samples was detected only in stage 1 and stage 3 samples but not in stage 2 and stage 4 samples in comparison to normal samples (Figure 3D). Moreover, while *SCL6A20* expression was not upregulated in any of the stages in PAAD samples, only stage 4 STAD samples were found to be upregulated in *SCL6A20* expression in comparison to their normal control groups (Figure 3E,F). In addition, *SLC6A20* mRNA expression was upregulated in all of the stages in THCA samples and in stages 1, 2, and 3 in UCEC samples compared to their normal counterparts (Figure S1A, B). Collectively, these findings indicated that *SCL6A20* expression might predict high stage risk in COAD, READ, ESCA, STAD, THCA, and UCEC patients.

### *SLC6A20* Expression Is Correlated with ACE2, TMPRSS2, and TMPRSS4

**Figure 4:**
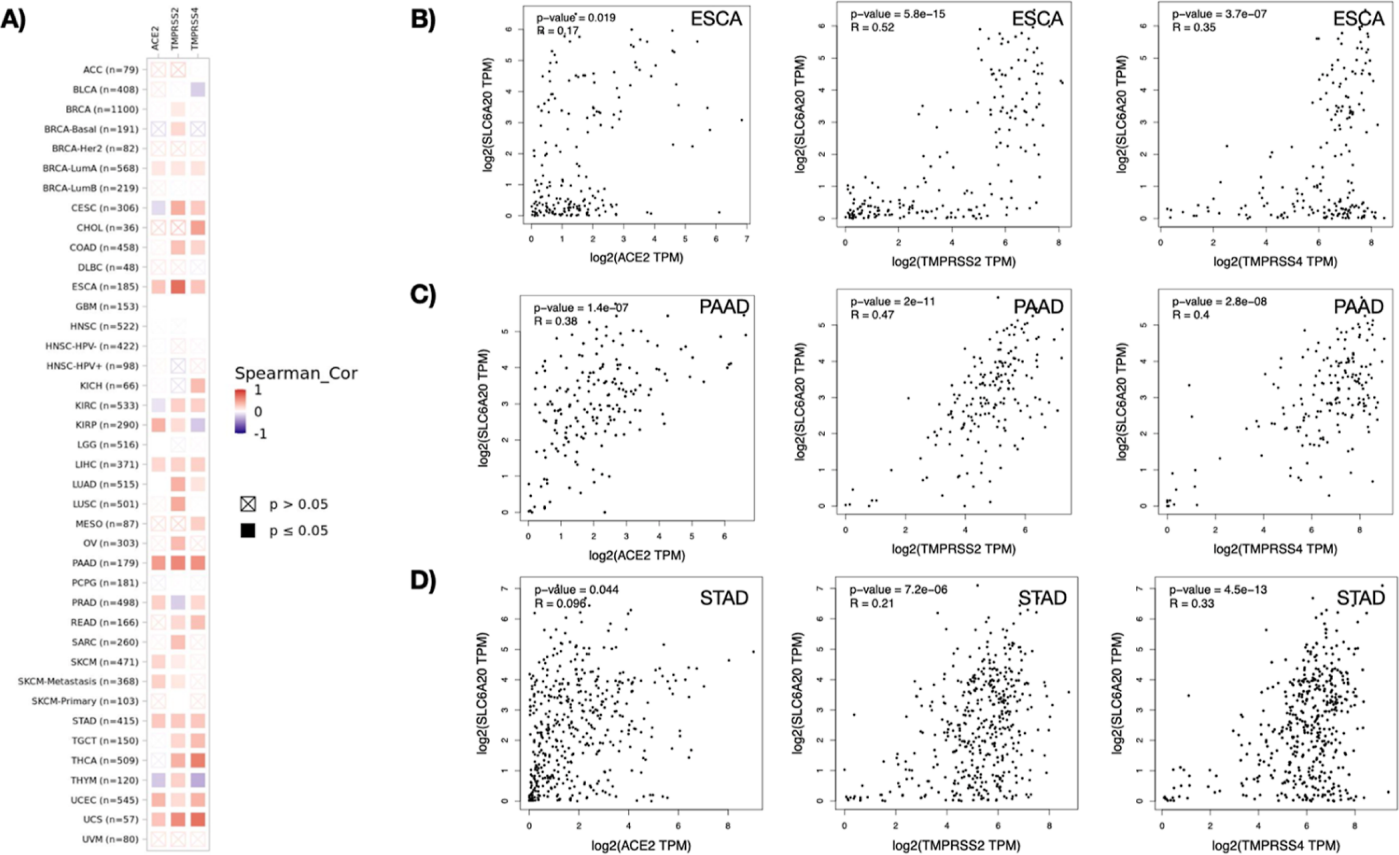
(A) Correlation analysis between *SLC6A20* and ACE2, TMPRSS2, and TMPRSS4 genes in pan-cancer samples. Scatter plot showing the correlation analysis between *SLC6A20* and ACE2, TMPRSS2, and TMPRSS4 in (B) ESCA, (C) PAAD, (D) STAD samples.

Given the prominent roles of ACE2, TMPRSS2, and TMPRSS4 in SARS-CoV-2 infection, the relationship between these genes and *SLC6A20* expression was investigated. The TIMER2.0 database^25^ was utilized for the assessment of co-expression of *SLC6A20* with ACE2, TMPRSS2, and TMPRSS4 in TCGA pan-cancer samples and showed that among the gastrointestinal cancers, ESCA, PAAD, and STAD together with UCEC and UCS exhibited positive correlation with *SLC6A20* gene expression (Figure 4A). This result was corroborated through the analysis of TGCA tumor and normal samples using the Gene Expression Profiling Interactive Analysis (GEPIA) database.^26^ The GEPIA database demonstrated that *SLC6A20* in ESCA samples was correlated with ACE2 (weak, *R* = 0.17, *p* = 0.019), TMPRSS2 (moderate, *R* = 0.52, *p* = 5.8 × 10^–15^), and TMPRSS4 (weak, *R* = 0.35, *p* = 3.7 × 10^–7^) (Figure 4B). For PAAD samples, *SLC6A20* was positively correlated with ACE2 (weak, *R* = 0.38, *p* = 1.4 × 10^–7^), TMPRSS2 (moderate, *R* = 0.47, *p* = 2 × 10^–11^), and TMPRSS4 (moderate, *R* = 0.4, *p* = 2.8 × 10^–8^) (Figure 4C). For STAD samples, *SLC6A20* expression was correlated weakly with ACE2 (weak, *R* = 0.096, *p* = 0.044), TMPRSS2 (weak, *R* = 0.21, *p* = 7.2 × 10^–6^), and moderately with TMPRSS4 (moderate, *R* = 0.33, *p* = 4.5 × 10^–13^) (Figure 4D). Additionally, in COAD samples, *SLC6A20* was positively correlated with ACE2 (weak, *R* = 0.26, *p* = 2.3 × 10^–11^), TMPRSS2 (weak, *R* = 0.1, *p* = 0.011), and TMPRSS4 (weak, *R* = 0.37, *p* = 0) (Figure S2A). Moreover, *SLC6A20* expression was correlated positively with ACE2 (moderate, *R* = 0.45, *p* = 0) and negatively with TMPRSS2 (weak, *R* = −0.23, *p* = 1.4 × 10^–5^) and TMPRSS4 (very weak, *R* = −0.087, *p* = 0.1) in KIRP samples (Figure S2B). Collectively, these results indicated that *SLC6A20* was positively correlated with SARS-CoV-2 infection genes ACE2, TMPRSS2, and TMPRSS4 mostly in gastrointestinal tumors, namely ESCA, PAAD, and STAD.

### *SLC6A20* Expression Is Detected in Innate and Adaptive Immune Cells

**Figure 5:**
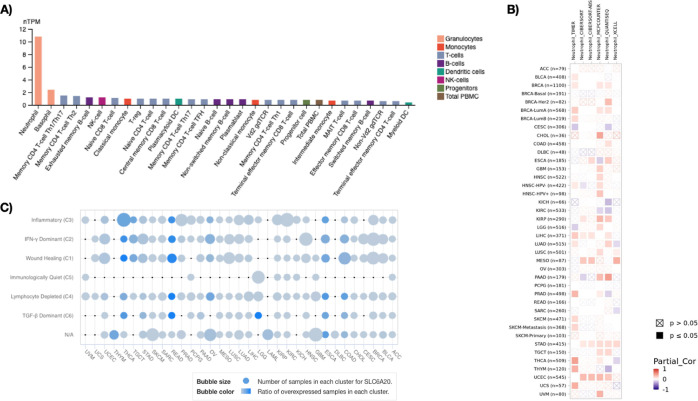
(A) *SLC6A20* expression in human immune cells in the Monaco data set. (B) Correlation analysis of *SLC6A20* expression in pan-cancer samples with neutrophil infiltration. (C) Number of samples in each cluster and ratio of *SLC6A20* expression in immune landscape of pan-cancer tumors.

Given that the COVID-19 disease is linked with a cytokine storm and inflammatory associated complications,^27^ the expression of *SLC6A20* gene was investigated in various immune cells. The Monaco data set showed that neutrophils and basophils exhibited the highest levels of *SLC6A20* expression, while the rest of the innate and adaptive immune cells had detectable amounts of *SLC6A20* expression (Figure 5A). Since *SLC6A20* expression was detected at the highest level in neutrophils, TGCA pan-cancer tumor samples were further examined using a TIMER2.0 data set. A correlation analysis using this data set to investigate a relationship of *SLC6A20* expression with neutrophil infiltration levels was performed. This analysis demonstrated a significant correlation of *SLC6A20* with neutrophil infiltration in a variety of tumors including ESCA, LIHC, PAAD, PRAD, BRCA, LUAD, KIRP, MESO, STAD, and UCEC (Figure 5B). To support the link between *SLC6A20* expression and COVID-19, the canSAR data set, providing information about the immune landscape of pan-cancer tumors, was utilized.^28^ This analysis demonstrated the association of increased *SLC6A20* expression with interferon-λ (IFN-λ), an immune related marker, and the inflammation signature particularly in THYM, READ, COAD, ESCA, and OV samples (Figure 5C). Given that IFN-λ acts as an independent risk factor in COVID-19 infection^29^ and inflammation is strongly associated with COVID-19 disease,^27^ this result suggests a contribution of *SLC6A20* expression for COVID-19 infection. Taken together, these findings indicate that elevated *SLC6A20* expression might play a role in immune infiltration in a variety of human tumor samples.

### PPI Network Analysis of *SLC6A20* and Pan-Cancer Assessment of TMEM27 Expression

**Figure 6:**
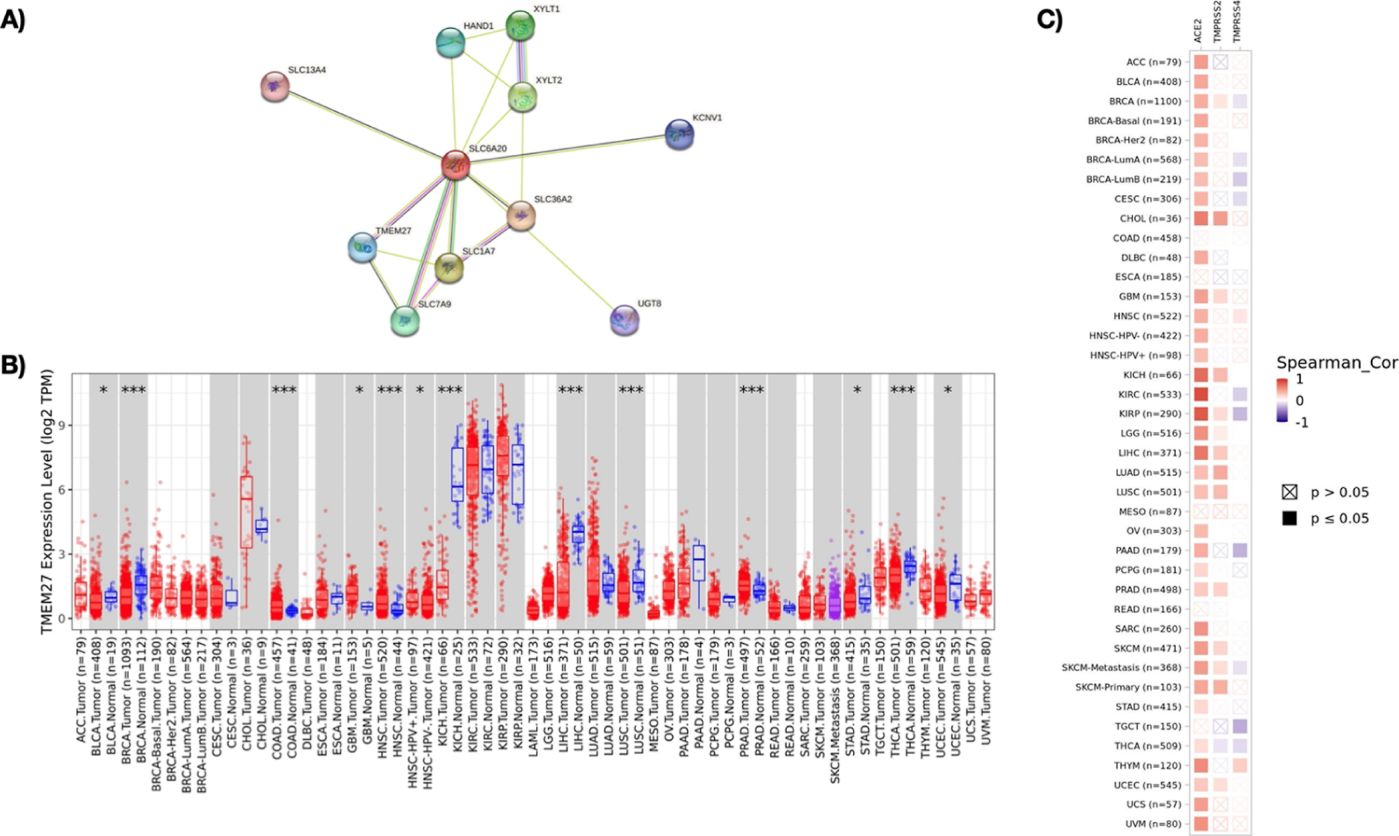
(A) PPI network analysis of *SLC6A20*-enriched proteins. (B) TMEM27 mRNA expression in pan-cancer tumor samples with normal counterparts. (C) Correlation analysis between TMEM27 and ACE2, TMPRSS2, and TMPRSS4 genes in pan-cancer samples.

To investigate the interplay between *SLC6A20* and its associated proteins, protein–protein interaction (PPI) network analysis was constructed using the STRING database.^30^ This analysis revealed a total of 10 expected edges with PPI enrichment p-value of 0.016 (Figure 6A). Most of the remaining *SLC6A20*-associated proteins in the same analysis included SLC family genes, SLC13A4, SLC7A9, SLC1A7, and SLC36A2 and additional proteins such as HAND1, XYLT2, KCNV1, and UGT8 (Figure 6A). Among the list of proteins interacting with *SLC6A20*, TMEM27 (collectrin), a homologue of ACE2 gene,^31^ was identified. Next, the involvement of TMEM27 was assessed in pan-cancer tumor samples using the UALCAN data set. This analysis revealed that TMEM27 expression was upregulated in BLCA, COAD, GBM, HNSC, LIHC, LUSC, PRAD, STAD, and THCA samples in comparison to their normal counterparts (Figure 6B). Furthermore, the interplay between TMEM27 and SARS-CoV-2 infection genes ACE2, TMPRSS2, and TMPRSS4 was investigated using the TIMER2.0 database. The correlation analysis performed using this database demonstrated that TMEM27 was positively correlated with ACE2 in all malignancies except COAD, ESCA, MESA, READ, and TGCT (Figure 6C). The same correlation analysis demonstrated that TMEM27 expression was positively correlated with TMPRSS2 in CHOL, GBM, KICH, PRAD, KIRP, LGG, LIHC, LUAD, LUSC, SKCM, BRCA, and UCEC (Figure 6C). The same analysis exhibited a negative correlation between TMEM27 and TMPRSS4 in a number of pan-cancer samples including KIRC, KIRP, PAAD, SKCM, TGCT, and THCA, while there was a weak positive correlation in HSNC and THYM samples (Figure 6C). Taken together, these findings indicated a potential involvement of *SLC6A20* with COVID-19 susceptibility in different types of human cancers via the association between *SLC6A20* and the ACE2 homologue TMEM27.

## Discussion

Rapid increase in the SARS-CoV-2 infection rates worldwide resulted in an immediate response to understand the underlying mechanisms of the COVID-19 disease, including in cancer patients. Cancer patients are considered as more prone to develop COVID-19 especially because of the impaired immune system response seen in them against the SARS-CoV-2 vaccine.^32,33^ Studies demonstrated that ACE2, TMPRSS2, and TMPRSS4 expression were upregulated and facilitated SARS-CoV-2 infection in tumor samples.^14,34^ In addition, certain host-specific genetic factors have been shown to play a critical role in increased susceptibility to COVID-19 in cancer patients.^35^ One of the causal genes identified to be linked with COVID-19 severity was *SLC6A20* gene located in the chromosome 3p21.31;^19,20^ however, its link with pan-cancer tumor samples remained to be elucidated. Here, we performed systematic analysis of *SLC6A20* expression in different malignancies and demonstrated that *SLC6A20* was upregulated in a wide number of human pan-cancer samples, and *SLC6A20* expression was associated with ACE2, TMPRSS2, and TMPRSS4 genes. In addition, our findings indicate that there is an interplay between *SLC6A20* expression and immune infiltration especially via increased amounts of neutrophils in a number of human tumor samples. Last, *SLC6A20* protein interacting with TMEM27, an ACE2 homologue, suggests the potential involvement of *SLC6A20* and TMEM27 in COVID-19 in cancer patients.

Two recent studies have characterized the 3p21.31 locus in the severity of COVID-19 disease.^19,20^ These studies utilized the genome and epigenome editing approaches to reveal COVID-19 causal genes regulated by the 3p21.31 locus. Among the genes regulated by this locus, *SLC6A20*, *CXCR6*, and *CCR9* were identified as causal genes to regulate COVID-19 risk in humans. Since *SLC6A20* gene was the only gene independently identified in these two studies for COVID-19 risk, we have focused on *SLC6A20* in human tumor samples to investigate *SLC6A20* expression in human pan-cancer samples and its association with SARS-CoV-2-related genes and immune infiltration. Our findings indicate that *SLC6A20* is highly expressed in a variety of human tumor samples, and there is a positive correlation with SARS-CoV-2 infection genes, ACE2, TMPRSS2, and TMPRSS4, suggesting that *SLC6A20* might be involved in modulating COVID-19 disease and hence increased COVID-19 susceptibility for cancer patients.

Subsequently, the relationship between *SLC6A20* and immune response in different malignancies was analyzed. The analysis demonstrated that *SLC6A20* was highly correlated with neutrophil infiltration in majority of pan-cancer samples including ESCA, KIRP, PAAD, PRAD, BRCA, LIHC, MESO, LUAD, STAD, and UCEC. As elevated immune infiltration is linked with prognosis of human cancers^36,37^ and found as an early indicator of COVID-19,^38,39^*SLC6A20* might be involved in modulating COVID-19 in cancer patients. Moreover, the positive association found between *SLC6A20* expression and neutrophil infiltration prompted us to think whether *SLC6A20* might be involved in regulating the cytokine storm in neutrophils as they are known to be one of the sources of cytokine release in COVID-19 patients.^40,41^ However, to support this claim, further functional studies will be needed.

In addition, immune subtypes inflammatory (C3), IFN-λ (C2), wound healing (C1), immunologically quiet (C5), lymphocyte depleted (C4), and TGF-β dominant (C6) analysis revealed an association between *SLC6A20*, particularly with inflammatory (C3) and IFN-λ (C2) signatures in THYM, READ, COAD, ESCA, and OV samples, suggesting a potential involvement of *SCL6A20* in immune modulation in these cancer types. Given that cancer and COVID-19 share common modalities in terms of being inflammatory^42,43^ and the role of IFN-λ^44,45^ in both processes, our findings suggest the potential involvement of *SCL6A20* in the pathophysiology of COVID-19 in cancer patients. Furthermore, efforts to treat severe COVID-19 patients with hypercytokinemia using emapalumab, an IFN-λ monoclonal antibody, is still underway with a registered clinical trial number of NCT04324021.

*SLC6A20* gene encodes the sodium-dependent imino transporter 1 (SIT1) protein.^46^ Importantly, it was reported that SIT1 was involved in viral entry and infection of SARS-CoV-2 in the human small intestine via heterodimerization with ACE2.^47^ Furthermore, SIT1 was reported to regulate glycine and proline transport.^48^ Moreover, *SLC6A20* knockout mice exhibited elevated plasma levels of glycine, suggesting the regulatory role of *SLC6A20* in glycine homeostasis.^49^ On the contrary, ACE2 knockout mice demonstrated decreased levels of plasma glycine concentration, suggesting the potential use of glycine itself in the treatment of COVID-19 patients.^50^ Indeed, it was proposed that glycine could intervene with anti-inflammatory cytokine storm seen in COVID-19 patients via interacting with its receptor GlyR to induce plasma membrane polarization and hence protection against the cytokine storm.^51^ To support this mechanism, one of the drugs used in the treatment of COVID-19 patients was ivermectin, an agonist for glycine-gated chlorine channels. While additional studies are still needed to clarify the antiviral effect of ivermectin in COVID-19 disease, a number of studies demonstrated ivermectin in the treatment of COVID-19.^52,53^

In this study, PPI enrichment analysis showed that TMEM27 was a *SLC6A20*-associated protein. TMEM27, a homologue of ACE2,^31^ has previously been reported as being co-regulated with ACE2 in bronchial samples.^54^ Given the prominent role of ACE2 in increased susceptibility in cancer patients and TMEM27 exhibiting 47% homology to ACE2 functions, we speculate that TMEM27 might be involved in the increased susceptibility in cancer patients for COVID-19. The evaluation of TMEM27 expression in pan-cancer samples resulted in elevated detection of TMEM27 mRNA expression in a number of cancer types such as COAD, GBM, HNSC, KIRC, KIRP, and PRAD. Alongside this observation, TMEM27 expression was found to be correlated predominantly with ACE2 and TMPRSS2 in pan-cancer samples, supporting the link between *SLC6A20* binding protein TMEM27 and SARS-CoV-2 infection-related proteins, ACE2 and TMPRSS2 in different cancer types.

## Conclusions

Collectively, our results suggest that *SLC6A20* might be involved in the increased susceptibility of cancer patients to COVID-19. *SLC6A20*, known to be regulated by the 3p31.21 locus and linked with the severity of COVID-19 disease, was further examined in pan-cancer samples. The analysis of pan-cancer tumor samples in comparison to their normal counterparts exhibited the upregulation of *SLC6A20* expression in a wide array of cancer types. In addition, *SLC6A20* expression was positively correlated with COVID-19-associated genes, ACE2, TMPRSS2, and TMPRSS4, in a number of tumor samples. Given the role of ACE2, TMPRSS2, and TMPRSS4 in providing increased susceptibility to COVID-19 in cancer patients, our findings indicate that *SLC6A20* might exhibit a similar function. Importantly, *SLC6A20* expression was associated with increased immune modulation particularly through gained IFN-λ and inflammatory signatures in different cancer types. Last, *SLC6A20* was found to interact with an ACE2 homologue protein TMEM27, and TMEM27 was upregulated in different cancer types along with its being positively correlated predominantly with ACE2 and TMPRSS2, which are known SARS-CoV-2-associated proteins. In conclusion, our study provides a systematic analysis of COVID-19 causal gene *SLC6A20* in pan-cancer samples.

## Materials and Methods

### Human Protein Atlas

The Human Protein Atlas is a web-based platform providing mRNA expression profiles of human genes. RNA-seq results obtained from 37 different types of human cancers and normal samples were visualized using the online available webpage.

### UALCAN Database

The UALCAN database was used to analyze *SLC6A20* gene expression in TCGA pan-cancer samples. The UALCAN database is an online webpage and provides an interactive environment to analyze the differential expression for a gene of interest in log 2 intensity in TCGA tumors including different pathological stages (stages 1, 2, 3, and 4).^22^

### HCCDB Database

The HCCDB is an interactive platform primarily for hepatocellular carcinoma but also includes RNA-seq data for pan-cancer tumors.^21^ Differential gene expression for *SLC6A20* in tumor and normal samples was analyzed in log 2 intensity.

### GEPIA Database

The GEPIA database provides an interface to perform gene expression correlation analysis between mRNA expression for selected genes using TCGA and normal samples.^26^ Scatter plots were generated using the interactive GEPIA database.

### TIMER2.0 Database

The TIMER2.0 database provides a systematic resource for cancer exploration and immune infiltrates in different malignancies in a webserver format.^25^ The cancer exploration module was utilized to determine the correlation coefficients for *SLC6A20* and ACE2, TMPRSS2, TMPRSS4 genes. In this analysis, the Spearman correlation was used, and when *p*-value was less than 0.05, it was significant. The immune gene module was used to determine the link with *SLC6A20* and infiltration of immune cells in different malignancies. The partial correlation (cor) and p-values were obtained using the Spearman rank correlation test.

### Acronyms

BLCA (bladder urothelial carcinoma), BRCA (breast invasive carcinoma), CHOL (cholangiocarcinoma), COAD (colon adenocarcinoma), ESCA (esophageal carcinoma), GBM (glioblastoma multiforme), HNSC (head and neck squamous cell carcinoma), KICH (kidney chromophobe), KIRC (kidney renal clear cell carcinoma), KIRP (kidney renal papillary cell carcinoma), LGG (low-grade glioma), LIHC (liver hepatocellular carcinoma), LUAD (lung adenocarcinoma), LUSC (lung squamous cell carcinoma), MESO (mesothelioma), OV (ovarian serous cystadenocarcinoma), PAAD (pancreatic adenocarcinoma), PRAD (prostate adenocarcinoma), READ (rectum adenocarcinoma), SKCM (skin cutaneous melanoma), STAD (stomach adenocarcinoma), TGCT (testicular germ cell tumors), THCA (thyroid carcinoma), UCS (uterine carcinosarcoma), UCEC (uterine corpus endometrial carcinoma).

### canSAR Database

The canSAR database is an interactive platform that enabled the integration of immune gene expression with *SLC6A20* expression.^28^ The database provided gene expression signatures for the following cellular functions: inflammatory (C3), IFN-λ (C2), wound healing (C1), immunologically quiet (C5), lymphocyte depleted (C4), and TGF-β dominant (C6).

### PPI Network

The STRING database was utilized to determine *SLC6A20* interacting proteins.^30^ Using this database, the protein network interacting with *SLC6A20* was identified.

### Statistical Analyses

The results obtained from various TCGA expression databases provided a *p*-value, whereby *p < 0.5, **p < 0.01, and ***p < 0.001 were considered as significant. *p*-values were derived from the databases as follows. The UALCAN database utilized Welch’s *T*-test for the significance between the difference in normal and tumor samples. The HCCDB database used the *t*-test function in *R* based on whether two groups had equal means or not followed by Benjamin–Hochberg correction. The UALCAN database performed *t*-test using a PERL script with the CPAN module. Pearson correlation analysis indicating ρ value: 0.00–0.19 (very weak), 0.20–0.39 (weak), 0.40–0.59 (moderate), 0.60–0.79 (strong), and 0.80–1.0 (very strong) was followed. *P*-value less than 0.5 was considered as significant. GEPIA webtool provided the Pearson correlation coefficients based on performing pair-wise gene expression correlation analysis.

## Supporting Information Available

mRNA expression levels of *SLC6A20* in different stages of TCGA tumor and the correlation analysis between *SLC6A20* and ACE2, TMPRSS2, and TMPRSS4 (PDF)

## References

[ref1] Chen Y.; Guo Y.; Pan Y.; Zhao Z. J. Structure Analysis of the Receptor Binding of 2019-NCoV. Biochem. Biophys. Res. Commun. 2020, 525, 135–140. 10.1016/j.bbrc.2020.02.071. 32081428 PMC7092824

[ref2] Walls A. C.; Park Y. J.; Tortorici M. A.; Wall A.; McGuire A. T.; Veesler D. Structure, Function, and Antigenicity of the SARS-CoV-2 Spike Glycoprotein. Cell 2020, 181, 281–292. 10.1016/j.cell.2020.02.058. 32155444 PMC7102599

[ref3] Shekaari A.; Jafari M. Structural Dynamics of COVID-19 Main Protease. J. Mol. Struct. 2021, 1223, 129235 10.1016/j.molstruc.2020.129235. 32929291 PMC7480992

[ref4] Stopsack K. H.; Mucci L. A.; Antonarakis E. S.; Nelson P. S.; Kantoff P. W. TMPRSS2 and COVID-19: Serendipity or Opportunity for Intervention?. Cancer Discovery 2020, 10, 779–782. 10.1158/2159-8290.CD-20-0451. 32276929 PMC7437472

[ref5] Sola I.; Almazán F.; Zúñiga S.; Enjuanes L. Continuous and Discontinuous RNA Synthesis in Coronaviruses. Annu. Rev. Virol. 2015, 2, 265–288. 10.1146/annurev-virology-100114-055218. 26958916 PMC6025776

[ref6] Harrison A. G.; Lin T.; Wang P. Mechanisms of SARS-CoV-2 Transmission and Pathogenesis. Trends Immunol. 2020, 41, 1100–1115. 10.1016/j.it.2020.10.004. 33132005 PMC7556779

[ref7] Grivennikov S. I.; Greten F. R.; Karin M. Immunity, Inflammation, and Cancer. Cell 2010, 140, 883–899. 10.1016/j.cell.2010.01.025. 20303878 PMC2866629

[ref8] Hanahan D.; Weinberg R. A. Hallmarks of Cancer: The next Generation. Cell 2011, 144, 646–674. 10.1016/j.cell.2011.02.013. 21376230

[ref9] Hanahan D. Hallmarks of Cancer: New Dimensions. Cancer Discovery 2022, 12, 31–46. 10.1158/2159-8290.CD-21-1059. 35022204

[ref10] Yalcin G. D.; Danisik N.; Baygin R. C.; Acar A. Systems Biology and Experimental Model Systems of Cancer. J. Pers. Med. 2020, 10, 180 10.3390/jpm10040180. 33086677 PMC7712848

[ref11] Yu J.; Ouyang W.; Chua M. L. K.; Xie C. SARS-CoV-2 Transmission in Patients with Cancer at a Tertiary Care Hospital in Wuhan, China. JAMA Oncol. 2020, 6, 1108 10.1001/jamaoncol.2020.0980. 32211820 PMC7097836

[ref12] Liang W.; Guan W.; Chen R.; Wang W.; Li J.; Xu K.; Li C.; Ai Q.; Lu W.; Liang H.; Li S.; He J. Cancer Patients in SARS-CoV-2 Infection: A Nationwide Analysis in China. Lancet Oncol. 2020, 21, 335–337. 10.1016/S1470-2045(20)30096-6. 32066541 PMC7159000

[ref13] Kerslake R.; Randeva H. S.; Jonigk D.; Werlein C.; Robertus J. L.; Katopodis P.; Jasker P.; Spandidos D. A.; Kyrou I.; Karteris E. Protein Expression of Transmembrane Protease Serine 4 in the Gastrointestinal Tract and in Healthy, Cancer, and SARS-CoV-2 Infected Lungs. Mol. Med. Rep. 2022, 25, 138 10.3892/mmr.2022.12654. 35211765 PMC8908323

[ref14] Temena M. A.; Acar A. Increased TRIM31 Gene Expression Is Positively Correlated with SARS-CoV-2 Associated Genes TMPRSS2 and TMPRSS4 in Gastrointestinal Cancers. Sci. Rep. 2022, 12, 11763 10.1038/s41598-022-15911-2. 35970857 PMC9378649

[ref15] Dai Y.-J.; Hu F.; Li H.; Huang H.-Y.; Wang D.-W.; Liang Y. A Profiling Analysis on the Receptor ACE2 Expression Reveals the Potential Risk of Different Type of Cancers Vulnerable to SARS-CoV-2 Infection. Ann. Transl. Med. 2020, 8, 481 10.21037/atm.2020.03.61. 32395525 PMC7210193

[ref16] Daniloski Z.; Jordan T. X.; Wessels H. H.; Hoagland D. A.; Kasela S.; Legut M.; Maniatis S.; Mimitou E. P.; Lu L.; Geller E.; Danziger O.; Rosenberg B. R.; Phatnani H.; Smibert P.; Lappalainen T.; tenOever B. R.; Sanjana N. E. Identification of Required Host Factors for SARS-CoV-2 Infection in Human Cells. Cell 2021, 184, 92–105. 10.1016/j.cell.2020.10.030. 33147445 PMC7584921

[ref17] Karim M.; Dunham I.; Ghoussaini I. Mining a GWAS of Severe Covid-19. N. Engl. J. Med. 2020, 383, 2588 10.1056/nejmc2025747. 33289971

[ref18] et al. Genomewide Association Study of Severe Covid-19 with Respiratory Failure. N. Engl. J. Med. 2020, 383, 1522–1534. 10.1056/nejmoa2020283. 32558485 PMC7315890

[ref19] Yao Y.; Ye F.; Li K.; Xu P.; Tan W.; Feng Q.; Rao S. Genome and Epigenome Editing Identify CCR9 and SLC6A20 as Target Genes at the 3p21.31 Locus Associated with Severe COVID-19. Signal Transduction Targeted Ther. 2021, 6, 85 10.1038/s41392-021-00519-1. PMC7897877 33619245

[ref20] Kasela S.; Daniloski Z.; Bollepalli S.; Jordan T. X.; tenOever B. R.; Sanjana N. E.; Lappalainen T. Integrative Approach Identifies SLC6A20 and CXCR6 as Putative Causal Genes for the COVID-19 GWAS Signal in the 3p21.31 Locus. Genome Biol. 2021, 22, 242 10.1186/s13059-021-02454-4. 34425859 PMC8381345

[ref21] Lian Q.; Wang S.; Zhang G.; Wang D.; Luo G.; Tang J.; Chen L.; Gu J. HCCDB: A Database of Hepatocellular Carcinoma Expression Atlas. Genomics, Proteomics Bioinf. 2018, 16, 269–275. 10.1016/j.gpb.2018.07.003. PMC6205074 30266410

[ref22] Chandrashekar D. S.; Karthikeyan S. K.; Korla P. K.; Patel H.; Shovon A. R.; Athar M.; Netto G. J.; Qin Z. S.; Kumar S.; Manne U.; Creighton C. J.; Varambally S. UALCAN An Update to the Integrated Cancer Data Analysis Platform. Neoplasia 2022, 25, 18–27. 10.1016/j.neo.2022.01.001. 35078134 PMC8788199

[ref23] Aznab M. Evaluation of COVID 19 Infection in 279 Cancer Patients Treated during a 90-Day Period in 2020 Pandemic. Int. J. Clin. Oncol. 2020, 25, 1581–1586. 10.1007/s10147-020-01734-6. 32654049 PMC7353830

[ref24] Wang B.; Huang Y. Which Type of Cancer Patients Are More Susceptible to the SARS-COX-2: Evidence from a Meta-Analysis and Bioinformatics Analysis. Crit. Rev. Oncol./Hematol. 2020, 153, 103032 10.1016/j.critrevonc.2020.103032. 32599375 PMC7295508

[ref25] Li T.; Fan J.; Wang B.; Traugh N.; Chen Q.; Liu J. S.; Li B.; Liu X. S. TIMER: A Web Server for Comprehensive Analysis of Tumor-Infiltrating Immune Cells. Cancer Res. 2017, 77, e108–e110. 10.1158/0008-5472.CAN-17-0307. 29092952 PMC6042652

[ref26] Tang Z.; Li C.; Kang B.; Gao G.; Li C.; Zhang Z. GEPIA: A Web Server for Cancer and Normal Gene Expression Profiling and Interactive Analyses. Nucleic Acids Res. 2017, 45, W98–W102. 10.1093/nar/gkx247. 28407145 PMC5570223

[ref27] Fara A.; Mitrev Z.; Rosalia R. A.; Assas B. M. Cytokine Storm and COVID-19: A Chronicle of pro-Inflammatory Cytokines. Open Biol. 2020, 10, 200160 10.1098/rsob.200160. 32961074 PMC7536084

[ref28] Mitsopoulos C.; di Micco P.; Fernandez E. V.; Dolciami D.; Holt E.; Mica I. L.; Coker E. A.; Tym J. E.; Campbell J.; Che K. H.; Ozer B.; Kannas C.; Antolin A. A.; Workman P.; Al-Lazikani B. CanSAR: Update to the Cancer Translational Research and Drug Discovery Knowledgebase. Nucleic Acids Res. 2021, 49, D1074–D1082. 10.1093/nar/gkaa1059. 33219674 PMC7778970

[ref29] Gadotti A. C.; de Castro Deus M.; Telles J. P.; Wind R.; Goes M.; Garcia Charello Ossoski R.; de Padua A. M.; de Noronha L.; Moreno-Amaral A.; Baena C. P.; Tuon F. F. IFN-γ Is an Independent Risk Factor Associated with Mortality in Patients with Moderate and Severe COVID-19 Infection. Virus Res. 2020, 289, 198171 10.1016/j.virusres.2020.198171. 32979474 PMC7510544

[ref30] Szklarczyk D.; Gable A. L.; Nastou K. C.; Lyon D.; Kirsch R.; Pyysalo S.; Doncheva N. T.; Legeay M.; Fang T.; Bork P.; Jensen L. J.; von Mering C. The STRING Database in 2021: Customizable Protein-Protein Networks, and Functional Characterization of User-Uploaded Gene/Measurement Sets. Nucleic Acids Res. 2021, 49, D605–D612. 10.1093/nar/gkaa1074. 33237311 PMC7779004

[ref31] Shin Y. H.; Jeong K.; Lee J.; Lee H. J.; Yim J.; Kim J.; Kim S.; Park S. B. Inhibition of ACE2-Spike Interaction by an ACE2 Binder Suppresses SARS-CoV-2 Entry. Angew. Chem., Int. Ed. 2022, 61, e202115695 10.1002/anie.202115695. PMC9011661 35043545

[ref32] Zeng C.; Evans J. P.; Reisinger S.; Woyach J.; Liscynesky C.; el Boghdadly Z.; Rubinstein M. P.; Chakravarthy K.; Saif L.; Oltz E. M.; Gumina R. J.; Shields P. G.; Li Z.; Liu S. L. Impaired Neutralizing Antibody Response to COVID-19 MRNA Vaccines in Cancer Patients. Cell Biosci. 2021, 11, 197 10.1186/s13578-021-00713-2. 34802457 PMC8606166

[ref33] Barrière J.; Chamorey E.; Adjtoutah Z.; Castelnau O.; Mahamat A.; Marco S.; Petit E.; Leysalle A.; Raimondi V.; Carles M. Impaired Immunogenicity of BNT162b2 Anti-SARS-CoV-2 Vaccine in Patients Treated for Solid Tumors. Ann. Oncol. 2021, 32, 1053–1055. 10.1016/j.annonc.2021.04.019. 33932508 PMC8080507

[ref34] Song J.; Han J.; Liu F.; Chen X.; Qian S.; Wang Y.; Jia Z.; Duan X.; Zhang X.; Zhu J. Systematic Analysis of Coronavirus Disease 2019 (COVID-19) Receptor ACE2 in Malignant Tumors: Pan-Cancer Analysis. Front. Mol. Biosci. 2020, 7, 569414 10.3389/fmolb.2020.569414. 33195415 PMC7649796

[ref35] SeyedAlinaghi S. A.; Mehrtak M.; MohsseniPour M.; Mirzapour P.; Barzegary A.; Habibi P.; Moradmand-Badie B.; Afsahi A. M.; Karimi A.; Heydari M.; Mehraeen E.; Dadras O.; Sabatier J. M.; Voltarelli F. Genetic Susceptibility of COVID-19: A Systematic Review of Current Evidence. Eur. J. Med.Res. 2021, 26, 46 10.1186/s40001-021-00516-8. 34016183 PMC8135169

[ref36] Shaul M. E.; Fridlender Z. G. Tumour-Associated Neutrophils in Patients with Cancer. Nat. Rev. Clin. Oncol. 2019, 16, 601–620. 10.1038/s41571-019-0222-4. 31160735

[ref37] Germann M.; Zangger N.; Sauvain M.; Sempoux C.; Bowler A. D.; Wirapati P.; Kandalaft L. E.; Delorenzi M.; Tejpar S.; Coukos G.; Radtke F. Neutrophils Suppress Tumor-infiltrating T Cells in Colon Cancer via Matrix Metalloproteinase-mediated Activation of TGF β. EMBO Mol. Med. 2020, 12, e10681 10.15252/emmm.201910681. 31793740 PMC6949488

[ref38] Prozan L.; Shusterman E.; Ablin J.; Mitelpunkt A.; Weiss-Meilik A.; Adler A.; Choshen G.; Kehat O. Prognostic Value of Neutrophil-to-Lymphocyte Ratio in COVID-19 Compared with Influenza and Respiratory Syncytial Virus Infection. Sci. Rep. 2021, 11, 21519 10.1038/s41598-021-00927-x. 34728719 PMC8563769

[ref39] Zhang B.; Zhou X.; Zhu C.; Song Y.; Feng F.; Qiu Y.; Feng J.; Jia Q.; Song Q.; Zhu B.; Wang J. Immune Phenotyping Based on the Neutrophil-to-Lymphocyte Ratio and IgG Level Predicts Disease Severity and Outcome for Patients With COVID-19. Front. Mol. Biosci. 2020, 7, 157 10.3389/fmolb.2020.00157. 32719810 PMC7350507

[ref40] Eijmael M.; Janssens N.; le Cessie S.; van Dooren Y.; Koster T.; Karim F. Coronavirus Disease 2019 and Peripheral Blood Eosinophil Counts: A Retrospective Study. Infection 2021, 49, 1325–1329. 10.1007/s15010-021-01710-w. 34625911 PMC8500265

[ref41] Zuo Y.; Yalavarthi S.; Shi H.; Gockman K.; Zuo M.; Madison J. A.; Blair C. N.; Weber A.; Barnes B. J.; Egeblad M.; Woods R. J.; Kanthi Y.; Knight J. S. Neutrophil Extracellular Traps in COVID-19. JCI Insight 2020, 5, e138999 10.1172/jci.insight.138999. 32329756 PMC7308057

[ref42] Turnquist C.; Ryan B. M.; Horikawa I.; Harris B. T.; Harris C. C. Cytokine Storms in Cancer and COVID-19. Cancer Cell 2020, 38, 598–601. 10.1016/j.ccell.2020.09.019. 33038939 PMC7531591

[ref43] Yang F.; Shi S.; Zhu J.; Shi J.; Dai K.; Chen X. Clinical Characteristics and Outcomes of Cancer Patients with COVID-19. J. Med. Virol. 2020, 92, 2067–2073. 10.1002/jmv.25972. 32369209

[ref44] Haji Abdolvahab M.; Moradi-kalbolandi S.; Zarei M.; Bose D.; Majidzadeh-A K.; Farahmand L. Potential Role of Interferons in Treating COVID-19 Patients. Int. Immunopharmacol. 2021, 90, 107171 10.1016/j.intimp.2020.107171. 33221168 PMC7608019

[ref45] Prokunina-Olsson L.; Alphonse N.; Dickenson R. E.; Durbin J. E.; Glenn J. S.; Hartmann R.; Kotenko S. v.; Lazear H. M.; O’Brien T. R.; Odendall C.; Onabajo O. O.; Piontkivska H.; Santer D. M.; Reich N. C.; Wack A.; Zanoni I. COVID-19 and Emerging Viral Infections: The Case for Interferon Lambda. J. Exp. Med. 2020, 217, e20200653 10.1084/jem.20200653. 32289152 PMC7155807

[ref46] Takanaga H.; Mackenzie B.; Suzuki Y.; Hediger M. A. Identification of Mammalian Proline Transporter SIT1 (SLC6A20) with Characteristics of Classical System Imino. J. Biol. Chem. 2005, 280, 8974–8984. 10.1074/jbc.M413027200. 15632147

[ref47] Camargo S. M. R.; Vuille-Dit-Bille R. N.; Meier C. F.; Verrey F. ACE2 and Gut Amino Acid Transport. Clinic. Sci. 2020, 134, 2823 10.1042/CS20200477. 33140827

[ref48] Semiz S. SIT1 Transporter as a Potential Novel Target in Treatment of COVID-19. Biomol. Concepts 2021, 12, 156–163. 10.1515/bmc-2021-0017. 34969185

[ref49] Bae M.; Roh J. D.; Kim Y.; Kim S. S.; Han H. M.; Yang E.; Kang H.; Lee S.; Kim J. Y.; Kang R.; Jung H.; Yoo T.; Kim H.; Kim D.; Oh H.; Han S.; Kim D.; Han J.; Bae Y. C.; Kim H.; Ahn S.; Chan A. M.; Lee D.; Kim J. W.; Kim E. SLC6A20 Transporter: A Novel Regulator of Brain Glycine Homeostasis and NMDAR Function. EMBO Mol. Med. 2021, 13, e12632 10.15252/emmm.202012632. 33428810 PMC7863395

[ref50] Singer D.; Camargo S. M. R.; Ramadan T.; Schäfer M.; Mariotta L.; Herzog B.; Huggel K.; Wolfer D.; Werner S.; Penninger J. M.; Verrey F. Defective Intestinal Amino Acid Absorption in Ace2 Null Mice. Am. J. Physiol.: Gastrointest. Liver Physiol. 2012, 303, G686–G695. 10.1152/ajpgi.00140.2012. 22790597

[ref51] Li C. Y. Can Glycine Mitigate COVID-19 Associated Tissue Damage and Cytokine Storm?. Radiat. Res. 2020, 194, 199 10.1667/RADE-20-00146.1. 32942307 PMC7574884

[ref52] Dinicolantonio J. J.; Barroso-Aranda J.; McCarty M. F. Anti-Inflammatory Activity of Ivermectin in Late-Stage COVID-19 May Reflect Activation of Systemic Glycine Receptors. Open Heart 2021, 8, e001655 10.1136/openhrt-2021-001655. 33875563 PMC8057070

[ref53] Barati N.; Motavallihaghi S.; Nikfar B.; Chaichian S.; Momtazi-Borojeni A. A. Potential Therapeutic Effects of Ivermectin in COVID-19. Exp. Biol. Med. 2022, 247, 1388–1396. 10.1177/15353702221099579. PMC9442455 35686662

[ref54] Aliee H.; Massip F.; Qi C.; Stella de Biase M.; van Nijnatten J.; Kersten E. T. G.; Kermani N. Z.; Khuder B.; Vonk J. M.; Vermeulen R. C. H.; Neighbors M.; Tew G. W.; Grimbaldeston M. A.; ten Hacken N. H. T.; Hu S.; Guo Y.; Zhang X.; Sun K.; Hiemstra P. S.; Ponder B. A.; Mäkelä M. J.; Malmström K.; Rintoul R. C.; Reyfman P. A.; Theis F. J.; Brandsma C. A.; Adcock I. M.; Timens W.; Xu C. J.; van den Berge M.; Schwarz R. F.; Koppelman G. H.; Nawijn M. C.; Faiz A. Determinants of Expression of SARS-CoV-2 Entry-Related Genes in Upper and Lower Airways. Allergy 2022, 77, 690–694. 10.1111/all.15152. 34698405 PMC8652715

