# Supplementary material for: Pan-Cancer Analysis of the COVID-19 Causal Gene SLC6A20
Source: ACS Omega. 2023 Mar 31;8(14):13153–61. doi: 10.1021/acsomega.3c00407 (PMC10081573; doi:10.1021/acsomega.3c00407)
Supplement: Supplementary file 1 — ao3c00407_si_001.pdf [file ao3c00407_si_001.pdf]

Pan-cancer analysis of the COVID-19 causal gene *SLC6A20*

Ahmet ACAR[1, *]

[1] Department of Biological Sciences, Middle East Technical University, Universiteler Mah. Dumlupınar Bulvarı 1, 06800 Çankaya, Ankara, Turkey

***Correspondence:** acara@metu.edu.tr

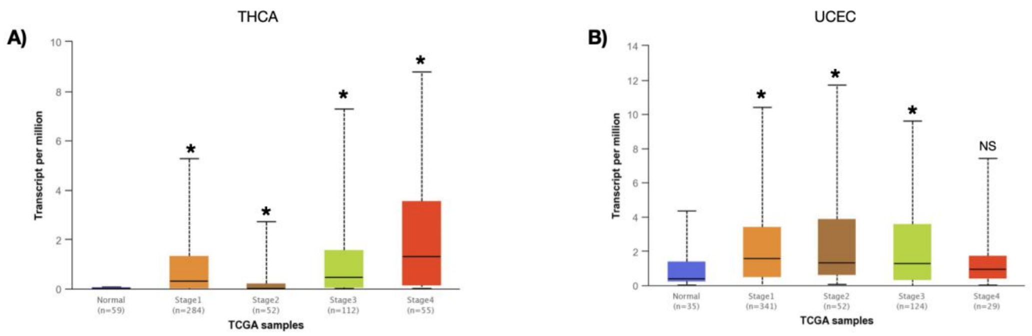

Figure S1 mRNA expression levels of *SLC6A20* in stage 1, 2, 3, and 4 TCGA and normal samples in **(A)** THCA, **(B)** UCEC.

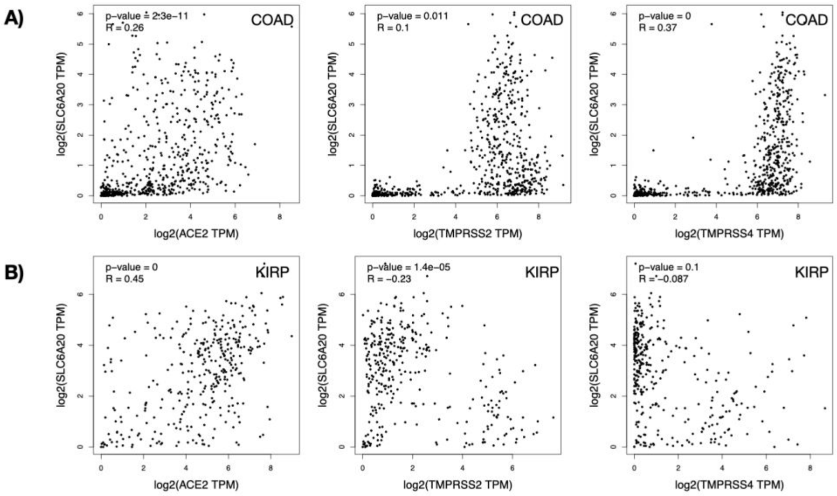

Figure S2 The correlation analysis between *SLC6A20* and *ACE2*, *TMPRSS2*, *TMPRSS4* provided via a scatter plot in **(A)** COAD, **(B)** KIRP samples.
